# A case report of Leptospirosis with positive cerebrospinal fluid targeted next-generation sequencing

**DOI:** 10.1016/j.idcr.2026.e02677

**Published:** 2026-07-07

**Authors:** X.X. Zhu, Y. Zhen, W.B. LI, X.H. Wang, F.L. Huang

**Affiliations:** a Department of Infectious Diseases, Affiliated Hospital of Southwest Medical University, Luzhou, China; b Department of Infectious Diseases, Fushun County People's Hospital, Fushun, China

**Keywords:** Leptospirosis, Icterohaemorrhagic type, Cerebrospinal fluid, Targeted next-generation sequencing, Multiple organ failure

## Abstract

Leptospirosis is a global zoonotic disease caused by pathogenic Leptospira species, predominantly Leptospira interrogans. Transmission to humans typically occurs via mucosal or percutaneous exposure to water contaminated by the urine of reservoir hosts, such as rodents and livestock. We report a case of a 67-year-old male admitted with a 2-day history of fever and a 1-day history of altered mental status, complicated by jaundice and hepatorenal failure. A definitive diagnosis of leptospirosis (icterohemorrhagic phenotype) was established by correlating the patient’s clinical manifestations and history of occupational exposure in rice paddies with the detection of Leptospira species via cerebrospinal fluid (CSF) targeted next-generation sequencing (tNGS).After treatment with meropenem, corticosteroids to prevent the Jarisch–Herxheimer reaction, and therapy to protect the liver and reduce jaundice, the patient’s organ function and mental status improved.This case suggests that for febrile patients with epidemiological exposure, neurological symptoms, and multi-organ involvement, early use of CSF tNGS can improve the diagnosis of leptospirosis, providing a critical basis for early and precise treatment.

## Introduction

Leptospirosis is distributed globally, with sporadic cases and outbreaks reported across most regions of China, particularly in the Yangtze River basin and southern provinces such as Sichuan, Hunan, and Jiangxi [1]. Surveillance data from Sichuan Province from 2004 to 2018 confirm that Icterohaemorrhagiae remains the stable and predominant circulating serogroup in China without strain replacement, while an average antibody seroprevalence of 24.52% in the healthy population suggests a degree of preexisting immunity alongside a persistent risk of infection [2]. Recently, the intensification of global climate change and the resulting increase in extreme weather events, such as flooding, have elevated the threat of leptospirosis outbreaks [3], [4]. High-risk populations include older male farmers [2], individuals with occupational exposure to rodents, and residents in areas with poor sanitation. Leptospirosis presents with a broad clinical spectrum, ranging from mild flu-like symptoms to life-threatening hepatic and renal failure or pulmonary hemorrhage, often leading to misdiagnosis due to symptomatic overlap with other tropical diseases like malaria and dengue [5]. Notably, a presentation with altered mental status as the initial symptom is relatively rare. We report a patient presenting with fever, jaundice, and altered mental status initially concerning for intracranial infection, in whom CSF tNGS rapidly confirmed leptospirosis, leading to a favorable prognosis after standard treatment. This case analysis aims to enhance clinical awareness of leptospirosis, particularly regarding its neurological manifestations, and underscores the importance of early pathogen identification and targeted therapy.

## Case presentation

**Fig. 1:**
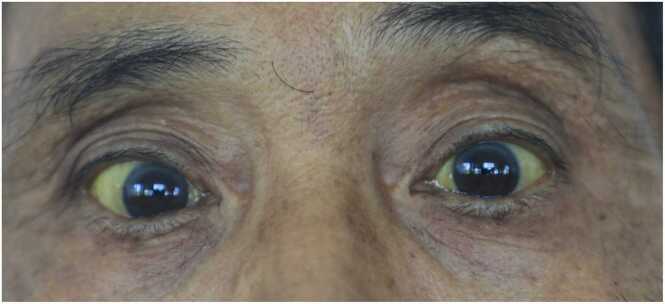
Conjunctival icterus in a patient with leptospirosis.

A 67-year-old man presented with a 2-day history of fever, a 1-day history of altered mental status, and generalized myalgia. Initial evaluation at another hospital revealed abnormal liver and kidney function. He had a history of working in a paddy field ten days before symptom onset and a past medical history of hepatitis with jaundice (already cured). Physical examination showed that the patient was alert, with generalized skin and scleral icterus (Fig. 1), barrel-shaped chest, reduced breath sounds in both lungs, soft and flat abdomen with muscle guarding, multiple areas of abdominal tenderness (predominantly in the right upper quadrant), and positive liver percussion pain. There was significant impairment of higher neurological function, rendering him unable to respond appropriately to questions. Muscle tenderness was present, deep tendon reflexes in the upper and lower limbs were decreased, and pathological reflexes were negative. Nuchal rigidity was positive, and Kernig signs were positive bilaterally. Laboratory tests revealed liver and kidney failure, thrombocytopenia, and elevated inflammatory markers (Table 1). Tests for respiratory viruses, urine culture, hepatitis virus antibodies, and Leptospira antibodies were unremarkable. Cranial imaging showed no specific abnormalities. The patient was admitted with a suspected diagnosis of intracranial infection. After admission, empirical therapy with cefoperazone-sulbactam was initiated, but on the same afternoon, due to persistently elevated inflammatory markers, the antibiotic was escalated to meropenem.Lumbar puncture revealed CSF pleocytosis with polymorphonuclear predominance and elevated protein, but normal glucose. Subsequent tNGS of the CSF identified Leptospira interrogans (351 reads; >50 copies/mL), with no other clinically significant pathogens detected.Correlating the epidemiological history, clinical presentation, and laboratory findings, a diagnosis of icterohemorrhagic leptospirosis was established. Under a regimen of meropenem, hepatoprotective and jaundice-reducing therapies, and dexamethasone to mitigate the Jarisch–Herxheimer reaction, the patient’s multiorgan function and mental status steadily improved without complications.

**Table 1:** Patient-related examination indicators.

Date	WBC	N%	HGB	PLT	PCT	CRP	BNP	Lac	AST
	(×10^9^/L)	(%)	(g/L)	(×10^9^/L)	(ng/mL)	(mg/L)	(pg/mL)	(mmol/L)	(U/L)
2025.8.23	5.93	71.84	117	44.4	22.94	174.70	821	0.64	N
2025.8.24	6.30	73.64	130	46.4	10.62	137.70	614	N	134
2025.8.25	5.55	73.04	126	44.4	5.27	83.50	234	N	72
2025.8.26	5.47	88.14	118	52.4	2.84	35.35	153	2.62	59
2025.8.27	6.06	75.64	125	882.4	N	N	N	N	68
2025.8.28	7.29	76.04	132	104.0	0.74	18.77	N	2.21	N
2025.8.30	18.26	85.11	130	202.0	0.34	85.79	N	N	63

Date	ALT	TBIL	DBIL	ALB	TBA	ALP	Crea	UA	DD	PTA
	(U/L)	(μmol/L)	(μmol/L)	(g/L)	(umol/L)	(U/L)	(umol/L)	(umol/L)	(ug/mL)	(%)
2025.8.23	N	N	N	N	N	N	648.5	1038	2.20	88.4
2025.8.24	77	208.70	191.07	29.9	N	N	665.4	963	2.17	89.8
2025.8.25	65	214.21	1988.03	27.8	303	94	433.6	631	2.69	N
2025.8.26	59	212.51	193.63	26.9	266	90	N	N	4.86	N
2025.8.27	60	167.12	156.69	24.4	214	100	156.3	269	N	N
2025.8.28	N	N	N	N	N	N	108.2	249	4.13	109.7
2025.8.30	64	83.58	74.06	28.4	47	105	77.3	178	2.27	87.0

Note:WBC,white blood cell; N%, neutrophil ratio; HGB, hemoglobin; PLT, platelet; PCT, procalcitonin; CRP, C-reactive protein; BNP, B-type natriuretic peptide; Lac, blood lactic acid; AST, aspartate aminotransferase; ALT, alanine aminotransferase; TBIL, total bilirubin; DBIL, direct bilirubin; ALB, albumin; TBA, total bile acid; ALP, alkaline phosphatase; Crea, serum creatinine; UA, uric acid; DD, D-dimer; PTA, prothrombin activity; N, not detected, data missing.

## Discussion

Epidemiological studies have demonstrated that geographic and climatic factors are key determinants of the distribution of leptospirosis, with a higher prevalence observed in flood-prone areas, subtropical regions, and rural settings [6]. The disease exhibits marked seasonality, with peak incidence reported from late August through the end of September [2]. The population at greatest risk comprises males aged 50–59 years [7]. The present patient was a 67-year-old male whose symptom onset occurred in late August, coinciding with the recognized peak transmission season. He reported occupational exposure to a paddy field ten days prior to symptom onset, representing direct contact with potentially contaminated water. These features are consistent with the established epidemiological profile of leptospirosis. The incubation period of leptospirosis ranges from 3 to 30 days [8]; the interval between exposure and symptom onset in this patient fell within this range, suggesting that the most likely route of infection was percutaneous inoculation through contact with paddy field water contaminated by rodent urine.

Leptospirosis shares overlapping clinical features with several other infectious diseases, including malaria, rickettsiosis, dengue fever, and hantavirus infection, rendering differential diagnosis challenging [9]. The clinical spectrum ranges from asymptomatic infection to the potentially fatal Weil's disease, which is characterized by the classic triad of jaundice, renal dysfunction, and hemorrhagic manifestations. Hepatic function typically recovers gradually following disease control, without long-term sequelae [6]. Although neurological manifestations are uncommon, they are heterogeneous and potentially serious, with aseptic meningitis representing the most frequently reported form [5]. The present patient exhibited fever, jaundice, acute renal failure, altered mental status, nuchal rigidity, and a positive Kernig's sign, a constellation of findings consistent with classic icteric leptospirosis (Weil's disease). CSF analysis revealed elevated white blood cell count (with a predominance of polymorphonuclear cells) and elevated protein level, which are consistent with the characteristics of intracranial infection caused by *Leptospira*.

The diagnosis of leptospirosis has long faced the challenge of a "gold standard that lags behind and insufficient sensitivity of conventional tests." Although the microscopic agglutination test (MAT) recommended by the WHO is the serological gold standard, it requires testing paired acute and convalescent serum samples (with a fourfold or greater increase in antibody titers) and takes up to 4–6 weeks, which cannot meet the need for early diagnosis [10]. Enzyme-linked immunosorbent assay (ELISA) for detecting IgM antibodies can shorten the turnaround time, but it has cross-reactivity with other spirochetal infections and may yield false-negative results when antibodies have not yet been produced in the early stage of illness (less than 7 days) [8], [11]. In this case, the patient's *Leptospira* antibodies were negative at admission, which is consistent with this issue. In contrast, molecular diagnostic techniques, such as PCR and targeted next-generation sequencing (tNGS), offer new approaches for early diagnosis. PCR can detect *Leptospira*-specific genes (e.g., *lipL32*) [12], but it requires primers designed for known targets and has limited ability to detect rare strains or mixed infections [12].PCR can detect Leptospira-specific genes (e.g., lipL32), but it requires primers designed for known targets and has limited ability to detect rare strains or mixed infections. In contrast, tNGS, which enriches for hundreds of clinically relevant pathogens through multiplex PCR amplification, offers a broader detection range than conventional PCR while maintaining a faster turnaround time and lower cost compared to unbiased metagenomic sequencing, making it practical for timely diagnosis in resource-limited settings. [13]. In this case, tNGS of cerebrospinal fluid was performed promptly after admission and detected *Leptospira interrogans* within three days, providing key evidence for the diagnosis. This finding echoes the classic 2014 report by Wilson et al. [5], in which a case of neuroleptospirosis was diagnosed by mNGS of cerebrospinal fluid after negative conventional tests. Together, these cases illustrate the clinical utility of advanced sequencing-based approaches in diagnosing central nervous system infections caused by rare pathogens.However, these techniques also have limitations, including inability to distinguish between live and dead bacteria, high cost, and technical demands.Therefore, they should not be used as first-line routine tests for leptospirosis. We recommend that these techniques be considered in the following settings:(1) patients with a clear epidemiological exposure history and clinical manifestations consistent with leptospirosis but negative conventional tests (MAT, ELISA, PCR); (2) patients with severe disease (e.g., multiorgan failure, central nervous system involvement) requiring rapid pathogen identification; and (3) suspected infection with rare or emerging *Leptospira* strains [14].

Regarding treatment, it is primarily divided into antibiotic therapy and supportive care. In the early stage, doxycycline or azithromycin is recommended as an initial antibiotic, while for patients with severe disease, penicillin G or ceftriaxone is suggested, as these agents can shorten the disease course and reduce the incidence of severe illness [15]. Previous reports have shown that meropenem combined with penicillin can be effective in treating leptospirosis complicated by multiorgan failure, with a favorable safety profile [16]. In addition, attention should be paid to the Jarisch-Herxheimer reaction, which occurs in approximately 21% of patients and manifests as fever and chills; it can be relieved by symptomatic fluid supplementation without the need to discontinue antibiotics [17]. In this case, the patient received empirical cefoperazone-sulbactam as anti-infective therapy upon admission because the etiology was unclear. On the same day, laboratory results returned indicating severe infection, leading to an escalation to meropenem. After seven days of anti-infective treatment, the patient's infection markers improved markedly, and liver and kidney function also improved. Dexamethasone was concurrently administered as anti-inflammatory therapy to prevent the Jarisch-Herxheimer reaction. Ultimately, both the patient's symptoms and laboratory parameters improved, and his condition stabilized.

In summary, for patients with a history of epidemiological exposure such as working in paddy fields or contact with floodwater, presenting with fever, jaundice, and multiorgan involvement — especially when the nervous system is affected — leptospirosis should be highly suspected. Early and comprehensive pathogen detection in cerebrospinal fluid or blood, combined with prompt and potent anti-infective therapy, prevention of the Jarisch-Herxheimer reaction, and multiorgan supportive care, can significantly improve patient outcomes. Clinicians should enhance their awareness of this disease, strengthen the collection of epidemiological history, and improve the use of pathogen detection to reduce the misdiagnosis rate and mortality.

## CRediT authorship contribution statement

**XiaoHong Wang:** Writing – review & editing. **WenBing Li:** Writing – review & editing, Data curation. **Yang Zhen:** Writing – review & editing, Data curation, Conceptualization. **XiXi Zhu:** Writing – review & editing, Writing – original draft, Conceptualization. **FuLi Huang:** Writing – review & editing, Writing – original draft, Supervision.

## Consent

Written informed consent was obtained from the patient for publication of this case report and accompanying images. A copy of the written consent is available for review by the Editor-in-Chief of this journal on request.

## Ethical approval

This study was approved by the Clinical Trial Ethics Committee of The Affiliated Hospital of Southwest Medical University (Approval No. KY2026295). The study was conducted in accordance with the principles of the Declaration of Helsinki.

## Funding

This research did not receive any specific grant from funding agencies in the public, commercial, or not-for-profit sectors.

## Declaration of Competing Interest

The authors declare that they have no known competing financial interests or personal relationships that could have appeared to influence the work reported in this paper.
